# Changes in body composition and plasma metabolites throughout lactation in high- and low-producing Saanen dairy goats

**DOI:** 10.1093/jas/skag006

**Published:** 2026-01-18

**Authors:** Jayde L Kirkham, Fernanda Zamuner, Alexander W N Cameron, Emmerson K Carpenter, Brian J Leury, Kristy DiGiacomo

**Affiliations:** School of Agriculture, Food and Ecosystem Sciences, Faculty of Science, The University of Melbourne, Parkville, VIC 3010, Australia; School of Agriculture, Food and Ecosystem Sciences, Faculty of Science, The University of Melbourne, Parkville, VIC 3010, Australia; Meredith Dairy Pty Ltd, Meredith, VIC 3333, Australia; Meredith Dairy Pty Ltd, Meredith, VIC 3333, Australia; School of Agriculture, Food and Ecosystem Sciences, Faculty of Science, The University of Melbourne, Parkville, VIC 3010, Australia; School of Agriculture, Food and Ecosystem Sciences, Faculty of Science, The University of Melbourne, Parkville, VIC 3010, Australia

**Keywords:** adipose tissue, body condition score, body mass index, dual energy X-ray absorptiometry, energy metabolism, sternum

## Abstract

This experiment investigated changes in body composition throughout lactation in dairy goats using non-invasive methods of body composition estimation including dual-energy X-ray absorptiometry (DEXA), body weight (BW), body condition score (BCS), and body mass index (BMI) in lactating Saanen dairy goats (20 high producers and 20 low producers). Body composition was measured on four occasions, early lactation (EL, 23 ± 6 DIM), mid lactation (ML, 107 ± 6 DIM), late lactation (LL, 206 ± 6 DIM), and 2 weeks post drying-off. The DEXA scans provide estimates of fat and lean tissue mass, and were evaluated by region (whole body, lower body, and sternal area). Data were analyzed using restricted maximum likelihood (REML) with stage of lactation and production group as fixed effects and goat as the random effect to account for repeated measurements within animals. Correlations among body composition measures were assessed using Pearson’s coefficients. Differences in body composition were driven primarily by stage of lactation rather than by production group. Milk yield was greatest in high producers during EL only (3.2 vs. 2.5 L/d; *P *< 0.05), but no differences were observed in energy corrected milk. The BW and BCS increased throughout lactation, peaking during the dry period. Changes in DEXA-derived fat and lean tissue mass were observed, with the largest change observed in sternal fat from EL to ML (−17%; *P *< 0.001). The BMI had stronger correlations with DEXA-derived body composition measures compared to BCS (*r* = 0.78 vs. 0.58; *P *< 0.001). Changes in composition were associated with changes in lactation stage rather than production level. While BW and BCS increased, overall, DEXA-derived fat mass decreased throughout lactation by 14% and increased by 10% from LL to the dry period (*P *< 0.001). To our knowledge, this is the first experiment to apply DEXA to measure body composition throughout lactation in goats. Findings suggest BMI may be a more reliable indicator of DEXA-derived body composition compared to BCS.

## Teaser

This experiment investigated changes in body composition throughout lactation in lactating dairy goats, revealing shifts in DEXA-derived fat reserves across lactation, with sternal fat identified as a key fat store.

## Introduction

During periods of negative energy balance, such as early lactation (EL) and late pregnancy, goats need to mobilize body reserves to sustain lactation. Understanding the mechanisms of mobilization and accretion of body reserves is important for determining the lifetime productivity and survival of livestock (Lerch et al., 2021). These insights can reveal differences between high-producing (HP) and low-producing (LP) animals, aiding in the development of targeted nutrition programs to support different stages of lactation. There is limited research investigating the changes in body reserves throughout lactation in dairy goats, and therefore, like other aspects of dairy goat production, research on dairy cows and sheep is often applied to goats. However, differences in composition and metabolism between goats and other dairy species limits the ability to reliably extrapolate findings across species (Stubbs and Abud, 2009; Zamuner et al., 2020). For example, the distribution of body fat in lactating dairy goats differs from ewes, as goats tend to store more fat in the sternum area rather than over the dorsal and lumbar regions (Carmichael et al., 2012).

Destructive measures of composition (e.g., chemical composition) are considered the “gold standard,” but these methods are not compatible with longitudinal studies as they require animal termination (Lerch et al., 2021). Previous research from Lerch et al. (2021) provided valuable insights into a direct calibration between chemical analysis and eight methods of body composition estimation in goats. Among the tools evaluated, computed tomography and deuterium oxide dilution space (D_2_OS) were the most accurate methods for in vivo empty-body composition analysis when compared to chemical composition (Lerch et al., 2021). However, both methods were time-consuming and expensive to measure. Therefore, there is a clear need to use faster and more efficient non-destructive methods to estimate body composition in larger groups of goats. Common measures of body composition analysis that can be easily implemented on-farm include live body weight (BW), body condition score (BCS) and body mass index (BMI). These measures are non-invasive, time effective, and do not require expensive equipment, although are also subjective, require restraint of the animals, and are prone to user errors (Lerch et al., 2021). For example, BCS was developed based on sheep, and involved palpation of the dorsal and lumbar regions (Mendizabal et al., 2011). However, in goats, palpation of the sternum region is also considered in the estimation of BCS, as previous research suggests goats tended to be more specialized in depositing fat subcutaneously, particularly in the sternal region (Mendizabal et al., 2007).

Dual energy X-ray absorptiometry (DEXA) is a rapid and non-destructive method for determining fat, lean tissue and bone mineral composition, based on the principle that X-rays passes through tissues at different intensities. Previous studies have demonstrated that DEXA is an accurate and reliable tool to determine body composition in live sheep (Hunter et al., 2011; Hunter et al., 2015; Miller et al., 2018), sheep half carcasses (Dunshea et al., 2007), lambs (Gardner et al., 2018), pigs (Suster et al., 2003), and beef and dairy carcasses (Calnan et al., 2021; Xavier et al., 2022). Furthermore, rapid DEXA scanner systems have recently been incorporated into some abattoirs, allowing real-time assessment of fat and lean composition at slaughter (Calnan et al., 2021). While no experiments have explored the use of rapid DEXA scans on live animals (without sedation), this technology presents an opportunity for future development of more practical methods for incorporating DEXA into commercial farming. Previous body composition research in goats have used tools such as computed tomography scans (Eknæs et al., 2017; Lerch et al., 2021), tritiated water kinetics (Dunshea et al., 1990), ultrasound of lumbar and sternum thickness (Knupp et al., 2021), and bioelectrical impedance analysis (Silva et al., 2018). However, to the best of our knowledge, there have been no studies using DEXA to determine the body composition of lactating dairy goats. However, strong correlations have been reported in live sheep. Hunter et al. (2011) demonstrated that DEXA-derived estimates of lean, fat, and bone mineral content were highly correlated with chemically determined composition in live animals (*R*^2^ > 0.97). In addition, DEXA has been used in goats to accurately determine the bone mineral density of trabecular bone after implants, supporting its precision and potential applicability for future in vivo research (Corten et al., 1997). These findings support the use of DEXA as a reliable, non-destructive tool for assessing body composition in small ruminants.

Metabolite concentrations in plasma, including glucose, urea and fatty acids, can be used as an indicator of nutritional status and energy balance in herds, particularly fatty acids which have been linked to ruminant performance and production in goats (Dunshea et al., 1989). This experiment aimed to investigate the changes that occur in dairy goat body composition and metabolic profiles throughout a lactation cycle using non-invasive measures of body composition estimation including DEXA, BW, BCS, BMI, and body measurements in HP and LP lactating dairy goats. We hypothesized that HP goats would mobilize a greater proportion of fat reserves during lactation compared to LP goats, particularly during EL.

## Materials and Methods

### Experimental facility

All experimental procedures were approved by the University of Melbourne Faculty of Veterinary and Agricultural Sciences Animal Ethics Committee (ID 25044). This experiment was conducted at Meredith Dairy commercial farm (Meredith, Australia, 37°50′S; 144°04′E).

### Animals, diet, and experimental design

Forty (40) second or third parity Saanen dairy goats, selected based on milk production in their previous lactation and grouped as either HP (*n* = 20) or LP (*n* = 20) were used in this experiment. Measurements, including body weight, BCS, BMI, milk and blood sampling, and DEXA scans, were performed over two consecutive days, on four occasions, during EL (23 ± 6 DIM), mid lactation (ML; 107 ± 6 DIM), and late lactation (LL; 206 ± 6 DIM), and 2 weeks post drying off (after approximately 290 days of lactation). Milk samples were only collected during lactation stages (i.e., not during dry period). The number of goats during each measurement period is shown in Table 1. Goats who had received medical intervention or died did not participate in the subsequent measurement periods. In addition, non-pregnant goats did not participate in the “dry” period measurements. Goats were housed with the regular milking herd in a one-sided, naturally ventilated shed that was north-south orientated with the open side facing east (approximately 500 per shed, stocking density approximately 1.5 m^2^ of floor space per head, 0.3 linear meters of feeder space per head, and straw bedding applied daily) throughout the experiment and fed a TMR ad libitum diet once daily at around 0700 hours. Approximate proportions (DM basis) of the components in the diet were barley (32%), lupins (25%), grass silage (27%), almond hulls (6%), and commercial pellets (10%; 20% CP, 13 MJ of ME/kg, 20% NDF, 26 g of Ca/kg, 12 g of P/kg). The same ration is fed to goats at all stages of lactation. Feed samples were collected for analysis once during each measurement period, and the average nutrient composition of the diet (per kg of DM) was 33% NDF, 17% CP, 12 MJ of ME. Goats were milked twice per day at approximately 0800 and 1600 hours in a 36-sided herringbone system fitted with automatic cup removers and in-line electronic milk meters (MidiLine SG Parlor, DeLaval Inc., Colac, VIC, Australia). Milk yield was automatically recorded twice daily.

**Table 1.** Fitted means of milk yield and components for high- and low-producing dairy goats measured at four stages of lactation: early (23 DIM), mid (107 DIM), late (206 DIM), and 2 weeks post-drying off (Dry)

	Early lactation			Mid lactation			Late lactation			Dry			SED^1^	*P*-value		
	High	Low	All	High	Low	All	High	Low	All	High	Low	All		*L*	*P*	*L* × *P*
***n***	20	20	40	20	19	39	15	16	31	15	14	29				
**BW, kg**	68	64	66^z^	71	67	69^y^	73	68	70^y^	80	73	76^x^	2.5	<0.001	0.17	0.44
**BCS**	2.5	2.5	2.5^z^	2.6	2.6	2.6^z^	2.8	2.7	2.8^y^	2.9	3.0	2.9^x^	0.07	<0.001	0.88	0.32
**Morphological measurements**																
** Height at withers, cm**	74	74	74^y^	74	74	74^y^	76	75	76^xy^	76	77	77^x^	1.4	<0.001	0.99	0.78
** Body length, cm**	81	80	81^y^	89	89	89^x^	88	89	88^x^	90	87	89^x^	1.6	<0.001	0.51	0.46
** Heart girth, cm**	94	93	94	96	95	95	97	96	97	99	92	96	2.1	0.12	0.20	0.07
** Sternum height, cm**	40	39	39	39	40	40	39	40	40	38	39	39	0.9	0.14	0.58	0.031
** Sternum thickness, mm**	37	36	36	39	37	38	50	47	48	49	47	48	2.0	<0.001	0.24	0.89
**BMI^2^**	0.91	0.87	0.89^y^	0.95	0.89	0.92^y^	0.95	0.90	0.92^y^	1.04	0.47	0.99^x^	0.017	<0.001	0.07	0.52

1 Standard error of difference (SED) represents the error associated with the interaction term (stage of lactation * production).

2 BMI refers to the BMI 2 equation: BMI 2 = BW/Height at withers.

x-z Means for L within a row that do not share a common superscript differ (*P* < 0.05).

*L*, stage of lactation; *P*, milk production.

### Dual-energy X-ray absorptiometry scanning

A Hologic Horizon W DEXA machine, in a portable, purpose-built truck, was used to scan the goats in this experiment (Hologic, MA, USA). To calibrate the DEXA machine, a spine phantom (supplied by Hologic and consisting of a known bone mineral content) was scanned on each experimental day before animal scans as outlined by Hunter et al. (2011). Goats were withheld from feed for approximately 12 h before scanning commenced. Goats were sequentially anesthetized (2.0 mg/10 kg BW xylazine and 100 mg/60–80 kg BW ketamine) and placed in a prone position with back legs extended and front legs placed back beside the trunk during the scan (approximately 13 min) as described by Hunter et al. (2011). Goats were scanned using the whole-body scan mode (APEX QDL software; Hologic, MA, USA; version 13.6.1.1). After scanning, goats were monitored in a recovery pen and fed once they were fully recovered.

**Figure 1.**
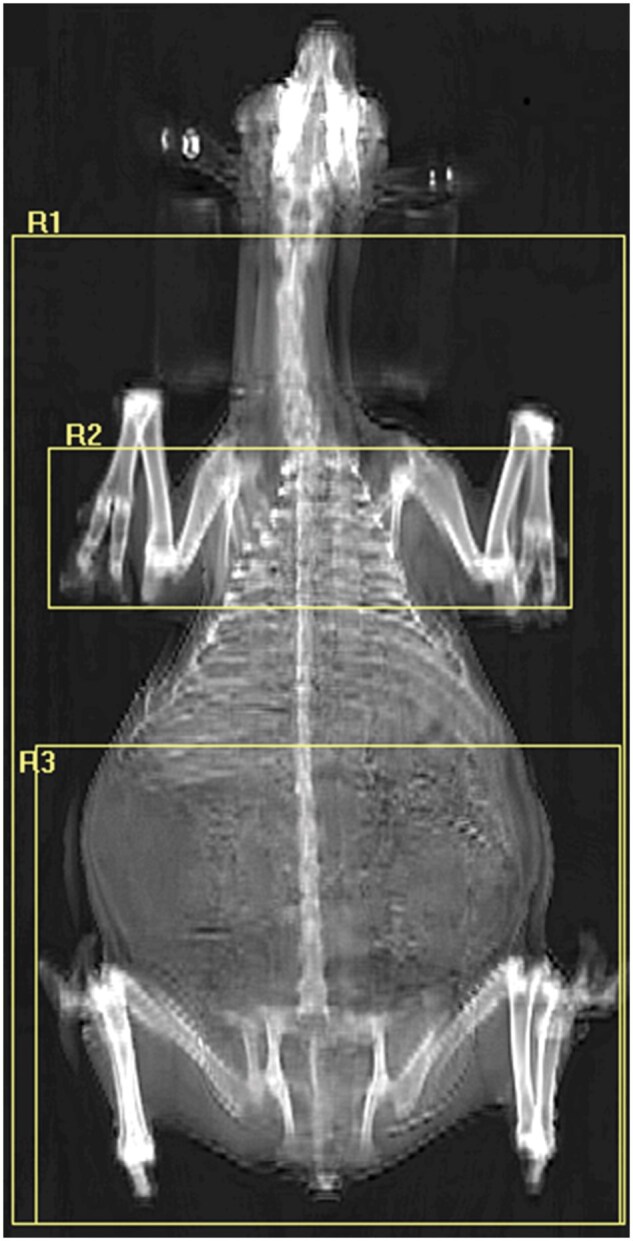
Dual energy X-ray absorptiometry regional analysis of animal scan image. R1 = whole body (excluding head), R2 = sternal region (first to sixth sternal rib), R3 = lower body including rumen (bottom of ribs to tail).

Measurements obtained from the DEXA machine include total tissue mass (TTM), lean tissue mass (LTM), fat tissue mass (FTM), and bone mineral composition. The APEX QDR software allows images to be analyzed in regions. An example of the regions used for analysis in this experiment are presented in Figure 1. Regions analyzed in this experiment included the whole body (excluding the head; WB), the sternal region (measured from the first to the sixth sternal rib and including the width of the body; SR), and the lower body (from bottom of rib cage to the tip of tail; LB). The WB region was selected as it most closely represented the “left arm” mode, which was described by Hunter et al. (2011) as the most precise analysis method. The additional two regions were proposed to represent the most likely regions for fat accretion in dairy goats.

### Milk and blood sampling

Individual milk samples were collected from the morning milking during all measurement periods (except dry), on the day prior to DEXA scanning. Samples were preserved with bronopol (Acticide L, Thor Chemicals Pty Ltd, NSW, Australia) and refrigerated at 4 °C until analysis in the laboratory at Herd Improvement Co-Operative (HICO; VIC, Australia) of milk fat, protein, and lactose using a NexGen milk analyzer (Bentley, MN, USA). Immediately after milking, and before feeding, blood samples (10 mL) were obtained via jugular venipuncture collected into blood tubes coated with lithium heparin. Blood was immediately stored on ice and centrifuged at (1,250 × *g*, 4 °C) for 10 min within 1 h of collection. Isolated plasma was stored at −20°C until analysis.

### Body weight, body condition score, and body measurements

On the day before DEXA scanning, individual live body weight was measured using a walk-over scale equipped with an electronic identification reader and weigh scale indicator (Tru-Test MP600 and Tru-Test XR5000; Tru-Test Livestock Management, VIC, Australia) after morning milking, and before the morning feed. Goats were weighed three times, and their average BW was used for accuracy. Individual BCS and morphological measurements were assessed on the same day as BW measurement. BCS evaluation was performed by the same two individuals using a 5-point scale based on visual assessment and palpation of the sternal and lumbar regions (Ghosh et al., 2019). Five morphological measures were taken while goats were standing in an upright position based on the methods described by Lerch et al. (2021). Briefly, we measured height-at-withers (withers; distance from the floor to the top of withers), heart girth (girth; behind the front legs and withers), body length (length; distance between the point of shoulder to the point of the pin bone), sternum height (distance from the floor to the bottom of sternum bone, measured in between front legs of the goat), and sternum thickness (measured by grasping each side of the sternum in between the front legs). Sternum thickness was measured using a manual body fat caliper. Height at wither and sternum height was measured using a measuring stick. A metric measuring tape was used for all other body measurements (refer to Supplementary Appendix S1 for diagram of measurement locations).

### Body mass index

There were six BMI equations tested in this experiment, based on Liu et al. (2019). The BMI was calculated as below:


$$BMI\;1-Girth=\text{BW}/\text{Girth(g}/{\text{cm}}^{2})$$

$$\mathrm{BMI}\;2-\mathrm{Withers}=\mathrm{BW}\;/\mathrm{Withers}(g/{\mathrm{cm}}^{2})$$

$$\mathrm{BMI}\;3-\mathrm{Length}=\mathrm{BW}\;/\mathrm{Length}(g/{\mathrm{cm}}^{2})$$

$$\mathrm{BMI}\;4-\mathrm{Withers}\;\times \;\mathrm{Length}=\frac{\mathrm{BW}}{\mathrm{Withers}\;\times \;\mathrm{Length}}(g/{\mathrm{cm}}^{2})$$

$$\mathrm{BMI}\;5-\mathrm{Girth}\;\times \;\mathrm{Length}=\frac{\mathrm{BW}}{\mathrm{Girth}\;\times \;\mathrm{Length}}(g/{\mathrm{cm}}^{2})$$

$$\mathrm{BMI}\;6-\mathrm{Withers}\;\times \;\mathrm{Girth}\;x\;\mathrm{Length}=\frac{\mathrm{BW}}{\mathrm{Withers}\;\times \;\mathrm{Girth}\;\times \;\mathrm{Length}}({\mathrm{cm}}^{3})$$

The equation for BMI 2 (including height at withers) was most highly correlated with measures of BCS and BW and was therefore the preferred equation used in this experiment (referred to as BMI). The Pearsons correlations between BMI equations, BCS and BW are shown in Supplementary Appendix S2. BMI 1–5 included measurements based on area, while BMI 6 included a volumetric approach to BMI. There was no significant benefit of including the volumetric approach to BMI and it had the lowest correlations with BCS and BW (*r* = 0.26 and 0.17, respectively; *P *< 0.05; Supplementary Appendix S2).

### Laboratory analysis

A portion of the whole blood sample was used to measure β-Hydroxybutyric acid (BHB) concentrations using a hand-held CentriVet Glucose and Ketone Reader (Pacific Vet, VIC, Australia) with bovine test strips, before plasma separation. At the time of the experiment, no BHB meters or strips had been validated specifically for goats or small ruminants; therefore, the bovine strips, which have been used in previous published literature, represented the most suitable available option (Kirkham et al., 2025). All plasma samples were analyzed in duplicate, and absorbance was measured on a Thermo Multiskanner plate reader (Thermo Fisher Scientific, Vantaa, Finland). Plasma fatty acids concentrations were measured using a commercially available kit (Wako C NEFA kit, Novachem, VIC, Australia; using the modified method by Johnson and Peters, 1993). Plasma glucose and blood urea nitrogen (BUN) concentrations were measured using commercially available colorimetric kits (Glucose GOD-POD, Thermo Scientific, Vantaa, Finland and QuantiChrom Urea Assay Kit, BioAssay Systems, CA, USA). Intra- and inter-assay coefficients of variation were less than 3.1% and <4.9% for fatty acids, <2.8% and <5.3% for glucose, <6.2% and <1.7% for BUN.

### Calculations and statistical analysis

Data was analyzed using GenStat (22nd Edition, VSN International LTD., Hemel Hampstead, UK), and RStudio (version 2024.04.2 Build 764, “Chocolate Cosmos” release, Posit Software, MA, USA), with R and Quarto for data visualization and reporting. Fitted means were estimated using Restricted Maximum Likelihood (REML) in GenStat, with stage of lactation (early, mid, late, dry) and production group (high, low) as fixed effects, and “goat” included as the random effect to account for repeated measurements on the same animal across lactation stages. After REML, pairwise comparisons of means were performed using the Bonferroni method to account for multiple comparisons. For SCC values only, a log10 transformation was applied, and the results presented represent the back-transformed means. Pearson’s correlation coefficients (*r*) were calculated and visualized using the “corrplot” package in R, and significance was calculated using the cor.mtest function in R to generate a matrix of *P*-values. Non-significant correlations (*P *> 0.05) were left blank to focus on meaningful relationships. Heatmaps were generated using the “pheatmap” package to illustrate correlations between metabolic and body composition variables. Figures, including line and bar plots, were created using ggplot2 package in R. *P*-values less than 0.05 were classified as significant.

## Results

**Figure 2.**
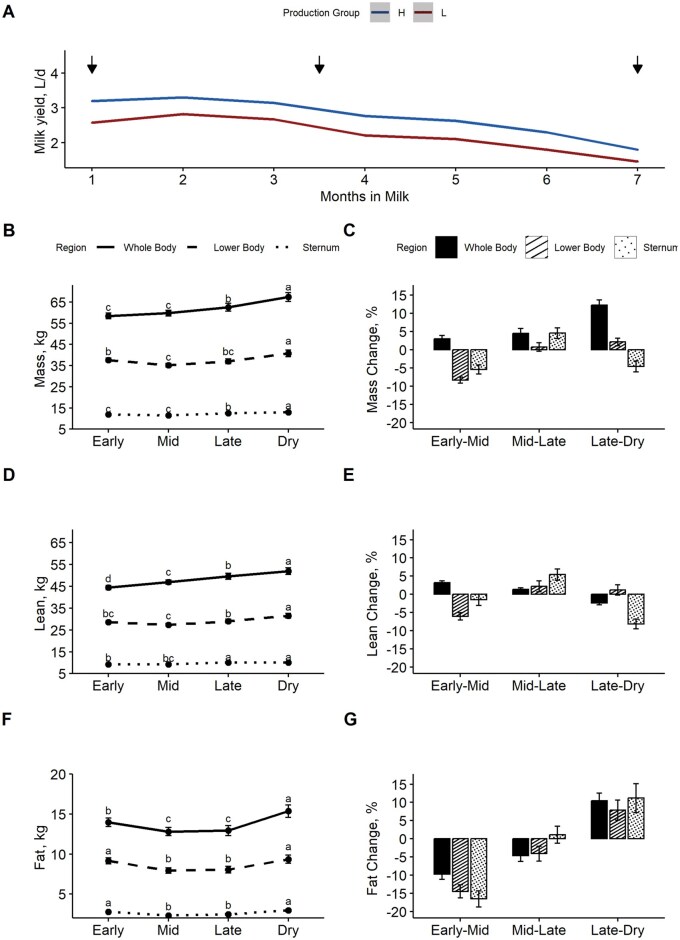
Changes in mass (B, C), lean (D, E), and fat (F, G) as determined by dual-energy X-ray absorptiometry (DEXA) across different regions and stages of lactation in dairy goats. Panel A shows milk production from 1 to 7 months in milk for high- and low-production groups, with arrows indicating the weeks in milk where DEXA scans were performed (early, mid, late). “Dry” refers to the last measurement period (2 weeks post drying off). Changes in total tissue mass (B, C), lean tissue mass (D, E), and fat tissue mass (F, G) are presented, with absolute changes on the left (in kg; B, D, F) and corresponding proportional changes (as a percentage of whole-body total mass) on the right (in %; C, E, G). Letters indicate significant differences across time for each region. Error bars represent standard error.

A summary of the physiological and production characteristics for the cohort of goats used in this experiment are presented in Tables 1 and 2, respectively. As expected, milk production was greatest during EL and decreased until the goats were dried off (Figure 2A). During EL, but not ML and LL, HP had significantly greater milk yield (L/d) compared to LP, but when converted to ECM (kg/d), there were no statistical differences observed between HP and LP groups (Table 2). There were also no differences observed in BW or BCS between HP and LP goats during any stage of lactation, despite some differences in milk yield and composition between HP and LP groups (Tables 1 and 2). Overall, changes in BW followed the same pattern in both groups, increasing from EL to ML, stabilizing between ML and LL, and reaching a peak in the dry period (Table 1). Similarly, overall BCS followed similar trends increasing from EL and ML to LL and the dry period (Table 1). The BCS was negatively correlated with milk yield and sternum thickness (*r* = −0.33 and −0.41, respectively; *P *< 0.05).

**Table 2.** Fitted means of body weight (BW), body condition score (BCS), morphological body measurements, and body mass index (BMI) for high- and low-producing dairy goats measured at four stages of lactation: early (23 DIM), mid (107 DIM), and late (206 DIM)

	Early lactation			Mid lactation			Late lactation			Production		SED^1^	*P-*value		
	High	Low	All	High	Low	All	High	Low	All	High	Low		*L*	*P*	*L* × *P*
***n***	20	20	40	20	19	39	15	16	31						
**Yield, L/d**	3.4^a^	2.7^b^	3.0^x^	2.8^b^	2.5^b^	2.6^y^	1.8^c^	1.5^c^	1.6^z^	2.7	2.2	0.17	<0.001	0.003	0.16
**Fat, %**	3.7	3.8	3.8	4.5	4.1	4.3	4.6	4.4	4.5	4.3	4.1	0.62	0.23	0.67	0.86
**Protein, %**	3.0	3.1	3.0^y^	3.1	3.1	3.1^y^	3.6	3.6	3.6^x^	3.2	3.3	0.14	<0.001	0.99	0.90
**Lactose, %**	4.7	4.6	4.7^x^	4.4	4.4	4.4^y^	4.5	4.5	4.5^xy^	4.5	4.5	0.13	0.007	0.85	0.56
**SCC, × 10^3^ cells/mL**	0.30	0.26	0.28^y^	0.91	0.51	0.68^x^	0.66	0.36	0.48^xy^	0.57	0.36	0.229	0.004	0.037	0.62
**ECM, kg/d**	3.4^a^	2.8^ab^	3.1^x^	3.0^a^	2.6^ab^	2.8^x^	2.1^bc^	1.7^c^	1.9^y^	2.9	2.4	0.28	<0.001	0.011	0.84

1 SED represents the error associated with the interaction term (*L* × *P*).

a-c L × P interaction means within a row that do not share a common superscript differ (*P* < 0.05).

x-z Means for L within a row that do not share a common superscript differ (*P* < 0.05).

ECM, energy corrected milk yield; *L*, stage of lactation; *P*, milk production; SCC, somatic cell count.

### Dual-energy X-ray absorptiometry measurements

**Figure 3.**
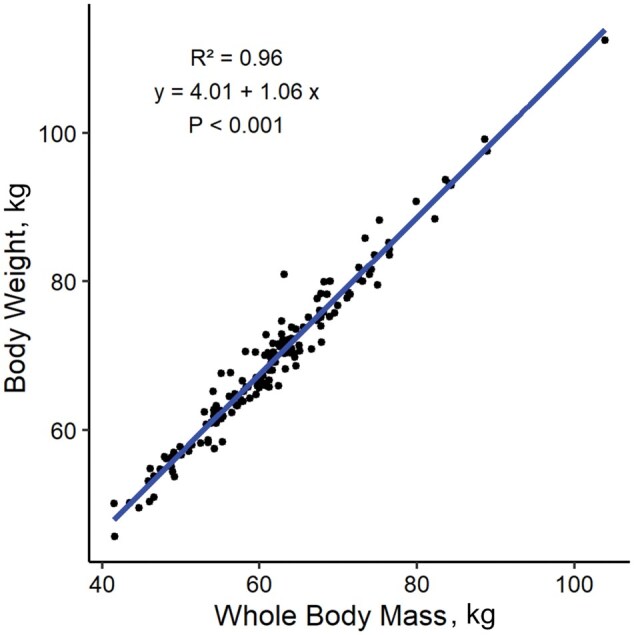
Linear regression analysis between whole-body mass derived from dual energy X-ray absorptiometry (DEXA), and body weight in dairy goats. The scatter plot displays individual data points, whilst the solid line represents the fitted regression model.

**Figure 4.**
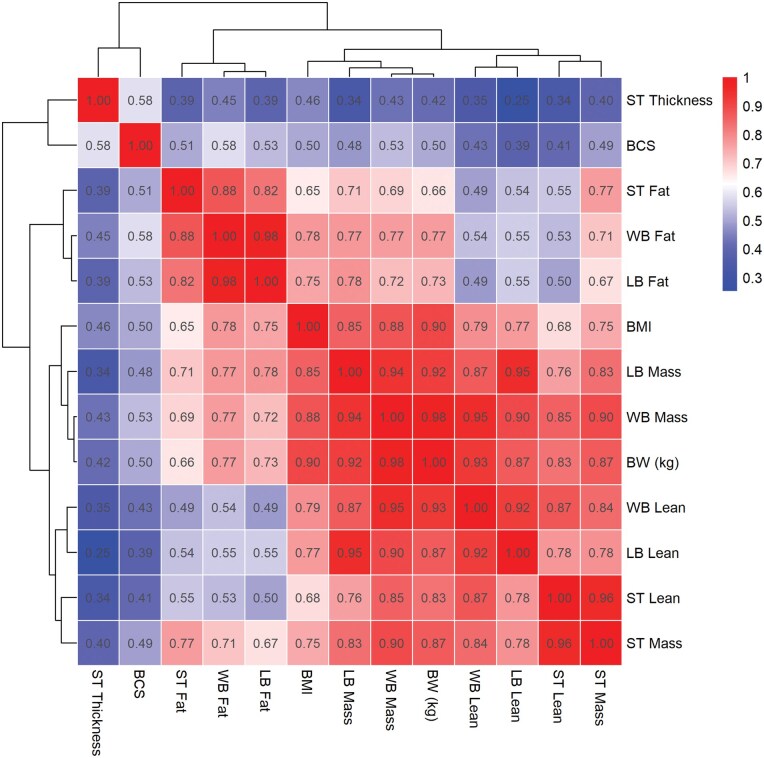
Heatmap of Pearson’s correlation coefficients (*r*) between different body composition components determined by dual-energy X-ray absorptiometry (DEXA) and other measures of body composition. A continuous colour gradient (see legend) encodes the magnitude and direction of the correlation. The dark red tiles represent strong positive correlations (*r* = 1), while the white tiles indicate a moderate correlation (*r* = 0.60), and dark blue indicate a low correlation (*r* = 0). Only significant correlations (*P *< 0.05) are displayed. The heatmap is clustered into groups based on their correlation similarity.

There were no differences observed between HP and LP goats for TTM, LTM, or FTM throughout lactation. The mean TTM, LTM, and FTM for each of the three regions analyzed during each stage of lactation are shown in Figure 2. In addition, Figure 2 shows the proportional change (as a percentage of WB) for each body region between stages of lactation compared to the previous measurement (i.e., proportional changes between EL to ML, ML to LL, and LL to Dry). The overall WB DEXA derived total mass increased progressively from EL to ML, LL, and Dry, as shown in Figure 2B. Briefly, Figure 2B shows that whole body FTM decreased from EL to ML and LL but was greatest during the Dry period. Because FTM and LTM are proportional components of TTM, an increase in FTM corresponds to a decrease in LTM. Sternum TTM was lowest during EL and ML, increased in LL, and reached its greatest mass during the Dry period (Figure 2F and G). The FTM showed the greatest proportional change from EL to ML, particularly in the sternal region which had a −17% change (*P *< 0.001) from EL to ML, and a + 10% change from LL to Dry. Overall, TTM for whole body was highly correlated with BW (*r* = 0.96, *P *< 0.001; Figure 3). The correlation between DEXA analysis measures and other composition measurements are shown in Figure 4. The BW was highly correlated with all DEXA measurements but showed the weakest correlations with sternum FTM, WB FTM, and LB FTM (*r* = 0.66, 0.77, and 0.73, respectively; *P *< 0.05; Figure 4).

### Circulating metabolites

**Figure 5.**
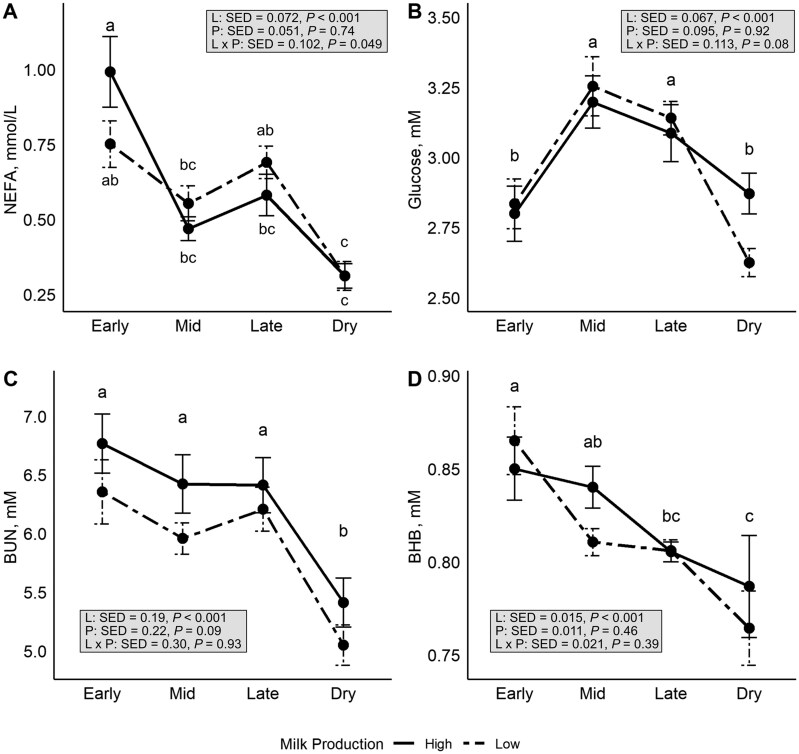
Least squares means (± standard error) for plasma concentrations of (A) non-esterified fatty acid (NEFA), (B) glucose, (C) blood urea nitrogen (BUN), and (D) β-Hydroxybutyric acid (BHB), measured across lactation stages (early-, mid-, late, and 2 weeks post drying off [non-lactating and pregnant]) in high- and low-producing dairy goats. Lines represent milk production groups (solid = high production; dashed = low production). Different letters indicate significant differences across lactation stages (*P *< 0.05).

Figure 5 illustrates the mean concentration of fatty acids for the HP and LP groups across the lactation stages. Fatty acids, BUN and BHB were greatest during EL, while glucose was lowest. The patterns of decline across lactation differed among metabolites, as illustrated in Figure 5. There were no differences in circulating fatty acids between HP and LP groups (*P *= 0.74), although the interaction term (between stage of lactation and production) was significant in this case (*P *= 0.049). The greatest change in fatty acids was evident in the HP group, which saw a large decline between EL and ML as seen in Figure 5A. Although no statistical difference, the HP and LP groups showed the largest divergence in fatty acids during EL which also corresponded to the largest divergence in ECM. Plasma BUN did not differ during EL, ML, or LL, but was lowest during Dry.

### Morphological measurements and body mass index

The morphological measurements and body mass index are presented in Table 1. The BMI was more highly correlated with all measures of composition (such as whole body, lower body, and sternal FTM, and BW), compared to BCS (Figure 4), except sternum thickness which both BMI and BCS had low correlations with.

## Discussion

This experiment investigated longitudinal changes in body composition throughout a lactation cycle in HP and LP dairy goats and evaluated the utility of non-destructive methods for estimating body composition including DEXA-derived estimates, BW, BCS, and BMI. Changes in body composition were evident at each lactation stage in both production groups, particularly between EL and ML, when milk yield gradually decreased, BW and BCS increased, and sternal fat declined by 17%. Given that circulating fatty acids increased while sternal fat decreased during this period, fat mobilization was likely elevated following early lactation. From ML to LL, goats maintained relatively stable BW, BCS, and DEXA-derived fat estimations. From LL to Dry, the second largest shift occurred, marked by increases in BW, BCS, and DEXA-derived fat, particularly in the sternal area (+10%). Overall, these patterns highlight the dynamic changes in body composition in HP and LP goats across the lactation cycle. Given that this experiment was conducted on a commercial farm, the selection of LP goats was constrained by management practices, as such animals are rarely retained. Consequently, differences between HP and LP goats were smaller than initially hypothesized, although this is consistent with the characteristics of the farm population.

To the best of our knowledge, this experiment is the first to successfully use DEXA to estimate body composition in live dairy goats. While DEXA has been extensively used in other livestock species, including sheep and cattle (Hunter et al., 2011; Miller et al., 2018; Calnan et al., 2021), its application in dairy goats has not previously been reported. Although DEXA has been widely used for body composition analysis in research and abattoir settings, it has certain limitations due to its reliance on X-ray attenuation and assumptions about body water content. In this experiment, goats were fasted (12 h) before DEXA scanning, to reduce variation in gut fill. However, given that small ruminants are difficult to fast completely (compared to a monogastric animal), there was likely still a degree of gut fill that could lead to overestimation of LTM in DEXA derived results. Similarly, Hunter et al. (2011) reported a 3% overestimation of LTM composition compared to chemical analysis, yet still found DEXA to provide highly accurate estimates of chemical composition in live sheep (Hunter et al., 2011). An important consideration when evaluating the practicality of utilizing DEXA technology is that animals are required to be immobile for the duration of the DEXA scan. Thus, anesthesia is essential and limits the feasibility for routine or large-scale monitoring of body composition in commercial herds. Instead, this technology is more suitably utilized in a research setting, where correlations between easier to implement, “on-farm” measured can be developed to ensure accurate assessment of composition in herds. On the other hand, in abattoirs, on-line DEXA methodology has been successfully validated with greater accuracy than existing carcass assessment methods in lambs, highlighting the potential for future on farm applications of this type of technology (Gardner et al., 2018). However, this would require further research and specific calibrations to implement.

### Correlations with non-destructive measures of composition

Understanding the mobilization and accretion of body reserves is important for determining lifetime productivity, estimating energy balance and improving production (Lerch et al., 2021). Both BW and BCS are commonly used measures of composition and assist farmers with estimating the status of a herd. In this experiment, while BW and BCS increased as lactation progressed, DEXA-derived measures of FTM decreased. In addition, BW had the lowest correlation with BCS compared to LTM and TTM, while BCS showed a moderate correlation with TTM, LTM, and FTM. In contrast, BMI exhibited greater correlations with all DEXA-derived measures of composition compared to BW and BCS, indicating that BCS may not be the most reliable indicator of DEXA-derived composition. The BMI equation included BW and height at withers. By incorporating height as a linear measure of frame size, this approach likely reduces the influence of short-term changes in body mass unrelated to tissue composition (e.g., gut fill), providing a more stable correlation with DEXA-derived body composition. There did not appear to be any advantage in using the alternative “volume”-based equation, consistent with the findings of Liu et al. (2019). Morphological measures alone did not provide a sufficient indication of body fat reserves when compared to DEXA-derived measures and BCS, but once combined into a BMI index, they were more useful as estimations of composition. Despite the inclusion of sternal palpation in the assessment of BCS, our results indicate only a moderate correlation with DEXA-derived sternal fat (*r* = 0.51), highlighting a limitation of manual scoring in estimation of body composition. Importantly, the weak correlation observed between manual BCS and DEXA-derived fat mass represents a significant limitation of current on-farm based body condition assessment practices. Our findings highlight that manual BCS lacks sufficient specificity and sensitivity to reliably reflect true body fat reserves. This limitation was emphasised in our experiment. While BW and BCS increased throughout lactation, DEXA-derived fat mass decreased, suggesting that manual BCS (including palpation of sternal region) may be confounded by changes in gut fill and visceral mass rather than adipose tissue. In contrast, the stronger and more consistent associations observed between BMI and DEXA-derived composition measures highlights BMI as a potentially superior and more robust field-based indicator of body composition. By incorporating height at withers as a proxy for skeletal frame size, BMI appears to mitigate the confounding effects of gut fill. These findings support the potential utility of BMI as a practical alternative to manual BCS for management of dairy goats, warranting further validation under commercial production conditions.

Recent studies in dairy cows have explored the development and validation of 3D imaging to provide an automated estimation of BCS, which is more time efficient, less subjective and potentially more accurate than manual BCS (Mullins et al., 2019; Martins et al., 2020; Albornoz et al., 2021). This technology would provide a practical way to monitor the body reserves of large groups of animals, without the need of restraint. Li and Teng (2022) utilized depth sensors to successfully measure different morphological points of both cattle and goats but did not investigate BCS. On the other hand, Lerch et al. (2021) utilized 3D technology which was originally developed for cattle, to predict BCS in goats, but found poor correlations between automated BCS and chemical composition (*R*^2^ ≤ 0.43). Further investigation and refinement to increase the accuracy of 3D BCS in goats could lead to more accurate, high-throughput assessments of body composition, with minimal human interference (Lerch et al., 2021).

### Early- and mid lactation

From EL to ML, BW, BCS and DEXA-derived WB mass increased for both production groups. Our results align with Cabiddu et al. (1999) who reported a negative correlation between BCS and milk yield (*r* = −0.24) in grazing dairy goats, likely due to the contrasting patterns of milk production and fat deposition. We also found a negative correlation between BCS and milk yield (*r* = −0.33) and between sternum thickness and milk yield (*r* = −0.41). As lactation progressed, milk yield decreased while BCS and sternum thickness increased. In addition, BCS and sternum thickness had a moderately positive correlation (*r* = 0.58), which can be explained by the inclusion of sternal palpation into the measurement of BCS in our experiment. However, BCS was more strongly correlated with DEXA-derived sternal fat than with sternum thickness (*r* = 0.51 vs. 0.39), although both correlations were only moderate. In our experiment, sternal thickness was measured using a manual caliper. Given that morphological measurements were taken whist the goats were standing and not under anesthetic, access to the sternal area was sometimes challenging. Alternative methods, such as measuring while the goats are sedated or using a digital caliper, could improve the accuracy of this measurement. Lerch et al. (2021) found that incorporating sternal palpation into BCS assessment provided a more accurate estimate of adipose tissues mass (*R*^2^ = 0.90). In our experiment, BCS increased from EL to LL, which is logical given that sternum thickness also increased as milk yield decreased. In contrast, Pazzola et al. (2011) observed a decline in BCS (including sternal palpation) in Sarda dairy goats as lactation progressed, although that could be attributed to different feeding strategies and herd management. Their goats were exclusively pasture-fed, without supplementary feeding, making them more prone to larger energy deficits compared to the intensively managed, TMR-fed goats in our experiment. Overall, our findings align with those of Cabiddu et al. (1999), demonstrating that BCS increases as lactation progresses, whilst also highlighting the influence of management and feeding strategies on BCS patterns across studies.

The DEXA whole body TTM was highly correlated with BW, which agrees with Hunter et al. (2011) who demonstrated that DEXA-derived measures of TTM, FTM and LTM were strongly related to BW and chemical composition in sheep. The greatest changes in DEXA-derived body composition were seen during EL. Whole body tissue mass (LTM and TTM) increased from EL to ML, whilst all DEXA-derived measures decreased in the lower body and sternum. Notably, the sternal area, considered a key site of fat deposition in dairy goats (Carmichael et al., 2012), showed a 16% decrease in FTM from EL to ML. The significant reduction in FTM suggests active mobilization of fat reserves, as expected, to support the energetic costs of peak lactation. Despite this period of fat mobilization, overall BW and BCS increased. This discrepancy suggests that BCS may not fully reflect changes in internal fat stores, as indicated by shifts in fat tissue mass derived from DEXA-derived measurements. This somewhat agrees with the findings of Lerch et al. (2021), who concluded that although not the best assessment of body composition, BCS is a useful, quick, and inexpensive estimate of composition. However, given the subjective nature of BCS, it is prone to user bias. In our experiment, BCS had the lowest correlations with DEXA-derived measures of fat for WB, LB and sternum (*r* = 0.58, 0.53, and 0.51, respectively), when compared to other non-destructive measures. On the other hand, BMI can be used as a more objective measure of composition compared to BCS, while being a more accurate indicator of DEXA-derived composition compared to BW (Liu et al., 2019). Given the limitations of DEXA for routine use in commercial settings, BMI, showing stronger correlations with DEXA-derived estimated than BCS, could serve as a more practical indicator of body composition. Validating these measures against DEXA in a research setting allows their application on-farm, bridging the gap between precise compositional assessment and routine herd management. Recently, Li and Teng (2022) investigated the use of cameras and deep learning technology to accurately determine various body measurements in goats and cows, including height at withers. Further development of these deep learning systems could facilitate the routine implementation of BMI on large-scale commercial farm. While BCS and BMI are relatively quick to perform, they both require restraint. Camera-based systems offer a rapid, non-invasive alternative that could overcome several of the limitations associated with traditional BCS.

During EL, there were high plasma concentrations of fatty acids and BHB, and low concentrations of glucose, further suggesting that fat mobilization was occurring during these early stages of lactation, whilst simultaneously goats were increasing BW and BCS overall. There were no differences in circulating fatty acids between HP and LP goats observed. Fatty acid concentrations of around 0.2 mmol/L in lactating animals can represent a neutral energy balance (Dunshea et al., 1989). At all points during lactation in this experiment, goats were above this neutral threshold. Plasma concentrations of fatty acids, glucose, and BHB were similar to Zamuner et al. (2020), who examined the endocrine and metabolic status of Saanen dairy goats up to three weeks post-partum. Zamuner et al. (2020) found that high-producing goats (4.0 L/d) had a 34% greater concentration of circulating fatty acids compared to the lower-yielding goats (2.4 L/d). In our experiment, the magnitude of difference between the HP and LP groups was smaller (in EL, HP = 3.4 L/d vs. LP = 2.7 L/d), which could help explain the lack of difference in fatty acids observed between groups. It is important to note that BHB concentrations were measured using bovine-validated strips due to the lack of goat-specific validated assays, which may introduce minor proportional bias in absolute values. Consequently, conclusions were drawn based on relative differences and trends, minimizing the impact of this limitation. Further to this, BHB concentrations in our experiment were similar to previously published values in lactating goats. Future research that focuses on developing and validating BHB strips specifically for goats would be highly beneficial for goat-specific research. Lastly, there were no differences in any morphological measurements or BMI between HP and LP goats during EL or ML, though greater BMI in HP goats compared to LP goats was approaching significance (*P *= 0.07).

### Mid- to late-lactation

From ML to LL there was an increase in BW, BCS and sternum thickness, and a decrease in milk yield for the HP, while the LP sustained similar levels of milk production. The whole body and sternum TTM and LTM increased, while FTM remained at similar levels. There were no key differences in plasma metabolites observed for the HP compared to LP goats. Overall, BUN levels remained stable throughout EL, ML, or LL, with a decline only observed during the dry period. BUN is often associated with protein metabolism, where elevated levels can indicate excessive dietary protein intake, increased protein catabolism, or inefficient nitrogen utilization. The relatively stable BUN concentrations suggest that dietary protein met metabolic demands without excessive urea accumulation. These smaller changes could reflect a change in nutrient partitioning as goats overcome peak lactation and start moving toward rebuilding and recovering tissue and body reserves into mid and late lactation, and that fat reserves are no longer being depleted to sustain peak lactation.

### Late lactation to dry period

After milk production ceased, goats continued to increase BW and BCS, while sternum thickness remained similar to LL. During the Dry period, when goats were also pregnant, total mass (LTM + FTM) reached a peak, reflecting a shift from fat mobilization to deposition. This transition was accompanied by the lowest circulating concentrations of fatty acids, BUN and BHB, whilst glucose remained at concentrations observed in EL. Notably, the sternum showed the greatest increase in fat accumulation, with an 11% increase in FTM, highlighting its critical role in fat storage in preparation for the next lactation cycle.

## Conclusions

This experiment provides new insights into changes in body composition across lactation stages in dairy goats, showing evidence of fat mobilization during EL (particularly in the sternum region), despite concurrent increases in BW and BCS. These findings provide an essential initial step towards accurately quantifying the mobilization and deposition of body reserves in this species. Compared to BCS, BMI showed stronger correlations with DEXA-derived estimations of composition, suggesting it may be a more useful on-farm indicator of body reserves. While DEXA can provide valuable insights in a research setting, its application on a large-scale farm is impractical due to high costs, the need for sedation, and logistical challenges. Therefore, further research into the potential application of BMI as an alternative to BCS based on the promising relationship with DEXA-derived estimations of composition is warranted. Future experiments validating DEXA-derived estimates of composition to the gold standard method (chemical composition) are also warranted. Moreover, studies across different breeds and nutritional regimes will be important to determine the generalizability of these findings. In addition, the development of an automated 3D body condition scoring system could enhance objectivity and reduce user bias and error, improving the reliability of BCS assessments.

## Supplementary Material

skag006_Supplementary_Data

## Abbreviations:

BCS: body condition score
BHB: β-hydroxybutyrate
BMI: body mass index
BUN: blood urea nitrogen
BW: body weight
D_2_OS: deuterium oxide dilution space
DEXA: dual energy X-ray absorptiometry
EL: early lactation
FTM: fat tissue mass
HP: high producing
LB: lower body
LL: late lactation
LP: low producing
LTM: lean tissue mass
ML: mid lactation
REML: restricted maximum likelihood
SR: sternal region
TTM: total tissue mass
WB: whole body
