# Supplementary material for: Changes in body composition and plasma metabolites throughout lactation in high- and low-producing Saanen dairy goats
Source: J Anim Sci. 2026 Jan 18;104:skag006. doi: 10.1093/jas/skag006 (PMC13014165; doi:10.1093/jas/skag006)
Supplement: skag006_Supplementary_Data [file skag006_supplementary_data.zip › 11-Mar-2026_102659_Appendices.docx]

**Changes in body composition and plasma metabolites throughout lactation in high- and low- producing Saanen dairy goats determined by dual energy x-ray absorptiometry.**

J. L. Kirkham,*^1^ F. Zamuner,^1^ A. W. N. Cameron,^2^ E. K. Carpenter,^2^ B. J. Leury,^1^, K. DiGiacomo^1^

^1^ School of Agriculture, Food and Ecosystem Sciences, Faculty of Science - The University of Melbourne, Parkville, Victoria 3010, Australia

^2^Meredith Dairy Pty Ltd., 106 Cameron Rd, Meredith, Victoria 3333, Australia

*Corresponding author: Jayde L. Kirkham, +61 409 002 164, [kirkham@student.unimelb.edu.au](mailto:kirkham@student.unimelb.edu.au)

# Appendices

# Appendix 1. Morphological measurements taken on dairy goat to assess body composition.

# Appendix 2. Pearson’s correlations between different body mass index (BMI) equations with body condition score and weight.

# Appendix 1. Morphological measurements taken on dairy goat to assess body composition.


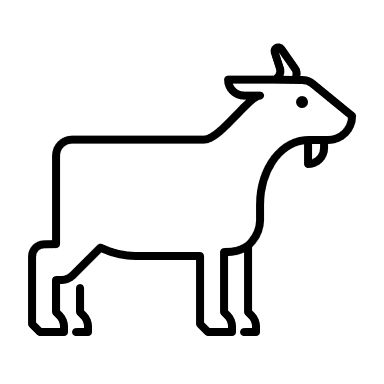


**1.**

**2.**

**3.**

**4.4.**

**5.4.**

**Appendix Figure A1.** Diagram of morphological measurements taken.

1. **Heart girth:** the circumference of the goat’s body, measured using a metric measuring tape. The measurement is taken just behind the front legs, wrapping around the body and over the point of the withers.

2. **Height-at-withers:** the distance from the floor to the highest point of the withers, measured using a measuring stick.

3. **Height-at-sternum:** the distance from the floor to the lowest part of the sternum (breastbone), measured between the front legs using a measuring stick.

4. **Body length:** the straight-line distance between the most forward point of the shoulder blade and the pin bone (near the tail), measured with a metric tape.

5. **Sternum thickness:** the sternum was measured by grasping each side of the breastbone directly between both front legs at the midline of the sternum. The measurement was taken twice in the same location using a manual caliper, ensuring even pressure was applied as the arrows on caliper aligned to record the measurement. The average of the two measurements was used as the final value.

# Appendix 2. Pearson’s correlations between different body mass index (BMI) equations with body condition score and weight.


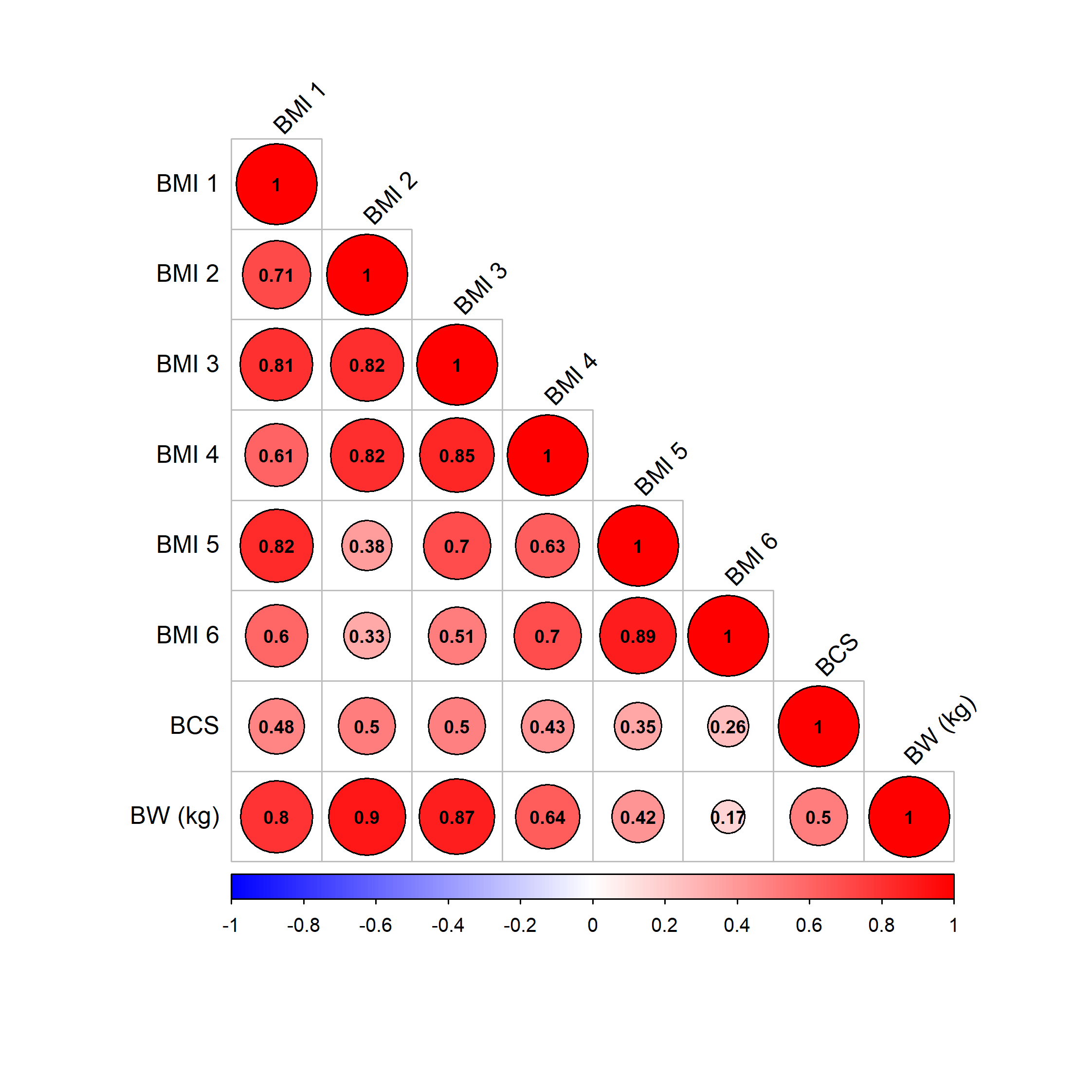


**Appendix Figure A2.** Pearson’s correlations between different body mass index (BMI) equations, with body condition score (BCS) and live body weight (BW). Only significant correlations are shows (*P* < 0.05). The bigger and darker the red circles, the closer the correlation is to 1. The smaller and darker the blue circles, the closer the correlation to -1.

Please refer to materials and methods section for BMI equations.
